# Prediction-error in the context of real social relationships modulates reward system activity

**DOI:** 10.3389/fnhum.2012.00218

**Published:** 2012-08-08

**Authors:** Joshua C. Poore, Jennifer H. Pfeifer, Elliot T. Berkman, Tristen K. Inagaki, Benjamin L. Welborn, Matthew D. Lieberman

**Affiliations:** ^1^Department of Psychology, University of California, Los AngelesLos Angeles, CA, USA; ^2^Department of Psychology, University of OregonEugene, OR, USA

**Keywords:** reward system, prediction-error, social reward, attachment, love, striatum, trust, fMRI

## Abstract

The human reward system is sensitive to both social (e.g., validation) and non-social rewards (e.g., money) and is likely integral for relationship development and reputation building. However, data is sparse on the question of whether implicit social reward processing meaningfully contributes to explicit social representations such as trust and attachment security in pre-existing relationships. This event-related fMRI experiment examined reward system prediction-error activity in response to a potent social reward—social validation—and this activity's relation to both attachment security and trust in the context of real romantic relationships. During the experiment, participants' expectations for their romantic partners' positive regard of them were confirmed (validated) or violated, in either positive or negative directions. Primary analyses were conducted using predefined regions of interest, the locations of which were taken from previously published research. Results indicate that activity for mid-brain and striatal reward system regions of interest was modulated by social reward expectation violation in ways consistent with prior research on reward prediction-error. Additionally, activity in the striatum during viewing of disconfirmatory information was associated with both increases in post-scan reports of attachment anxiety and decreases in post-scan trust, a finding that follows directly from representational models of attachment and trust.

## Introduction

The human reward system anticipates and monitors the acquisition of primary rewards such as food (Ikemoto, 2007), and conditioned rewards like money (Pessiglione et al., 2006). Yet, recent evidence suggests this system also responds to purely social rewards like altruistic behavior (Moll et al., 2006; Hare et al., 2010), verbal praise (Kirsch et al., 2003), approving faces (Rademacher et al., 2010), equitable treatment (Tabibnia et al., 2008; Tricomi et al., 2010), and reputational gains (Behrens et al., 2008). These findings and others suggest that the reward system, its integrated subcortical and cortical networks plays a pivotal role in the development of long-term social attachments (i.e., relationships), affiliative traits, and representation of specific social partners. Moreover, these findings also suggest that social representations learned through a process of associative learning similar to that which underlies basic stimulus-behavior conditioning and reinforcement (see Depue and Morrone-Strupinsky, 2005; Fehr and Camerer, 2007; Behrens et al., 2008; Lieberman and Eisenberger, 2009; Grabenhorst and Rolls, 2011; Lin et al., 2012).

Reward system regions, such as the ventral striatum and the ventral tegmental area (VTA), evidence a prediction-error signal consisting of fluctuations of activity in response to violations of expectations for potential rewards outcomes. The VTA and anterior portion of the ventral striatum (aVS) evidence this activity in response to both unexpected gains and omissions of rewards (Schultz, 2006; D'Ardenne et al., 2009), while more posterior portions of the ventral striatum (pVS) show this activity in response to unexpected losses (see Seymour et al., 2007). A wide body of research indicates that prediction-error activity largely provides the basis for the reward system's role in associative learning, and modulates activity in cortical regions such as the ventromedial prefrontal cortex (vmPFC), which in turn provide a representational basis for both the incentive qualia of external stimuli and their reliability as a source of reward (Depue and Morrone-Strupinsky, 2005; Ikemoto, 2007; van den Bos et al., 2007; see also Kahnt et al., 2010). Empirical work finds that activity within the VTA discriminates between images of romantic partners, friends, and strangers (Bartels and Zeki, 2000; Aron et al., 2005; Xu et al., 2010; Acevedo et al., 2012; see Diamnond and Dickenson, 2012 for review). Numerous other studies demonstrate reward system prediction-error signals under conditions of social reward-related reinforcement expectation violation (Behrens et al., 2008; Jones et al., 2011; see Fehr and Camerer, 2007 for reviews). Additionally, other studies similarly find signals related to social validation and rejection within cortical reward system projection sites such as the anterior cingulate cortext (Eisenberger et al., 2003; Somerville et al., 2006) and vmPFC (see Grabenhorst and Rolls, 2011; Lin et al., 2012). Collectively, a wide body of research finds evidence the mesocorticolimbic reward system and integrated cortical networks serves as a common valuation-learning system across classes of reward, including social rewards (see Fehr and Camerer, 2007 for review). However, evidence for reward system sensitivity to social rewards is largely limited to studies of strangers engaged in game-theoretic simulations of social interaction, studies wherein participants are evaluated by hypothetical peers, and studies of participants viewing images of their romantic partners. No study has yet examined reward system prediction-error in response to violations of participants' *a priori* social reward expectations *perpetrated by participants' actual relationship partners*. This leaves unclear the full extent to which theory about the reward-system's role in social cognition and relationship formation generalizes to day-to-day social life.

Additionally, research has not yet linked reward system activation in response to social feedback from specific individuals with changes in specific representations of those individuals (e.g., attachment). The extent to which models of reward-system mediated learning apply to the development of social representations and sentiments in a similar way that they do to behavioral outcomes and intuition (Lieberman, 2000) remains unclear, and data is limited with respect to whether social attachment representations, in particular, are *learned* through a process of social reward mediated, associative learning and valuation that is dependent on the mesocorticolimbic reward system. Nonetheless, there is reason to believe such links do exist (see Amodio and Frith, 2006; Vrticka et al., 2008; Insel, 2010). Attachment security (see Pierce and Lydon, 2001; Reis et al., 2004; Shaver and Mikulincer, 2006) and trust (Rempel et al., 1985) hinge on the *predictability*—moreso than positivity or negativity—with which specific romantic partners are responsive to self-related needs for esteem, validation, and care (Rempel et al., 1985; Reis et al., 2004; Eastwick and Finkel, 2008). Indeed, Attachment Theory (see Shaver and Mikulincer, 2006 for review) asserts that the conceptual attachment system dynamically regulates care-seeking behavior based on the reliability with which partners are responsive and that both globalized (across relationships) and partner specific models of attachment (within specific relationships; see Pierce and Lydon, 2001). Unpredictable partners engender insecure-anxious attachments (see Shaver and Mikulincer, 2006 for review) toward specific partners, characterized by *appetitive* partner-related seeking behaviors and rumination (Eastwick and Finkel, 2008). In this respect, attachment anxiety represents uncertainty about relationship partners, provides the motivational impetus for pursuing and engendering deeper commitments with relationship partners (Eastwick and Finkel, 2008), and coincides with feelings of intense romantic affect (romantic passion; Hatfield and Walster, 1978). Other research notes that this intense affect bears semblance to addiction-related phenomenology (Aron et al., 2005; see also Ortigue and Bianchi-Demicheli, 2008) and may be intimately tied to reward system functions (Hyman, 2005). Taken together, prevailing models of attachment development and phenomenology are remarkably reminicient to those of reward-system mediated learning.

Using an event-related fMRI paradigm in a sample of real romantic partners, we examined whether the reward system evidences prediction-error-like signals under conditions of social reward-related uncertainty owing to violations of participants' *a priori* self-reported expectations for their romantic partners' valuation of them on positive attributes (esteem; social-reward). Moreover, given extisting research and the similarities between attachment dynamics and reward-system processing, we expected that unpredictable violation and validation of individuals' expectations for their current partners' esteem of them (social reward) should be associated with increases in partner-specific attachment anxiety (uncertainty in specific relationships) and decreases in partner-specific trust (certainty in specific relationships). Furthermore, we expected that regional activity in key mesocorticolimbic reward system areas during expectation violations would be related to task-related reports of attachment anxiety, trust, and task-related affect, and whether activity owing to violations of in either positive or negative directions would have differential associations on these reports.

## Methods

### Sample

Participants were 17 right-handed individuals [nine women; Age (yrs.): *M* = 26.44; *SD* = 7.89] currently involved in a long-term romantic relationship [Relationship Length (mos.): *M* = 52.94; *SD* = 54.84], recruited through Craigslist postings. Participants' romantic partners provided supporting data. No participants reported MRI contraindications.

### Intake questionnaire

Prior to the lab session, participants and their partners completed online intake questionnaires in which they appraised how descriptive each of 100 positive attributes were of their partner and their relationship, as well as reported their expectations of how their partners' would appraise them on each of the items (see “Appendix” for complete list). Items originated from measures assessing commitment (Rusbult et al., 1998), partner responsiveness (Gable et al., 2006), partner preference (Fletcher et al., 1999), and partner investment (Ellis, 1998). Participants made appraisals with 7-point Likert scales [anchors: A Little (1), Exceedingly (7); mid-point: Moderately (4); *M* = 5.24, *SD* = 0.73, range = 3.12, skew: −0.08]. Participants also completed pre-task measures of relationship-specific attachment anxiety (after Brennan et al., 1998) and partner-specific trust, using 9-point [anchors: Not At All True (1), Strongly Agree (9)] and 7-point [anchors: Strongly Disagree (1), Strongly Agree (7)] Likert scales, respectively.

### Laboratory session

One week later, participants came to the lab and were told that they would receive “feedback” from their partners' appraisal questionnaires—statements similarly phrased to match items in the intake questionnaire, that would reflect their partners' reported appraisals of them (see “Appendix” for additional information). In reality, this feedback was based solely on participants' *expectations* of their partners' appraisals of them.

During the subsequent MRI session, functional scans were acquired while participants received feedback either confirming or violating their expectations about their partners' questionnaire responses, on a trial-by-trial basis (see “Appendix” for fMRI considerations). Participants received three different kinds of feedback: (1) confirmations of their expectations (i.e., exactly as expected), (2) positive violations of their expectations (i.e., better than expected), and (3) negative violations of their expectations (i.e., worse than expected). Positive and negative violations were operationalized as prediction-error events during this task, and were constructed by adding or subtracting two scale points from participants' responses to the reflected appraisal questionnaire. For example, if participants marked “VERY” (scale point: 5) to the item, “I think my partner thinks I am ____ kind,” a confirmation would be phrased, “I think (Participant Name) is VERY (scale point: 5) kind.” A positive violation would be phrased, “I think (Participant Name) is EXCEEDINGLY (scale point: 7) kind.” A negative violation would be phrased, “I think (Participant Name) is FAIRLY (scale point: 3) kind.” Participants were not shown scale numbers, but were aware of where the different labels fell on the scale due to extensive exposure to the scale prior to scanning. Items were randomly assigned to be either confirmations or violations.

Multiple efforts were made to ensure that participants believed the cover story and that they actually received feedback from their romantic partners. First, prior to the task, participants rated another “participant,” who would take part in the study at a later data, on the same intake questionnaire items and scale they used to appraise their partner. This exercise served the purpose of reacquainting participants with the scaling used in the intake appraisal questionnaires—in reality, there was no other participant. Second, the task began with 10 training trials, which included an audio recording of partners actually reading the statement out loud. Stimuli for training trials were selected from a pool of items wherein participants' expectations about their partners' responses to appraisal items were identical to their partners' actual appraisals of them (training trials were not included in analyses). In this way, partners' were not suspicious of the credibility of stimuli they were recording for subjects (see “Appendix” for more details).

**Figure 1:**
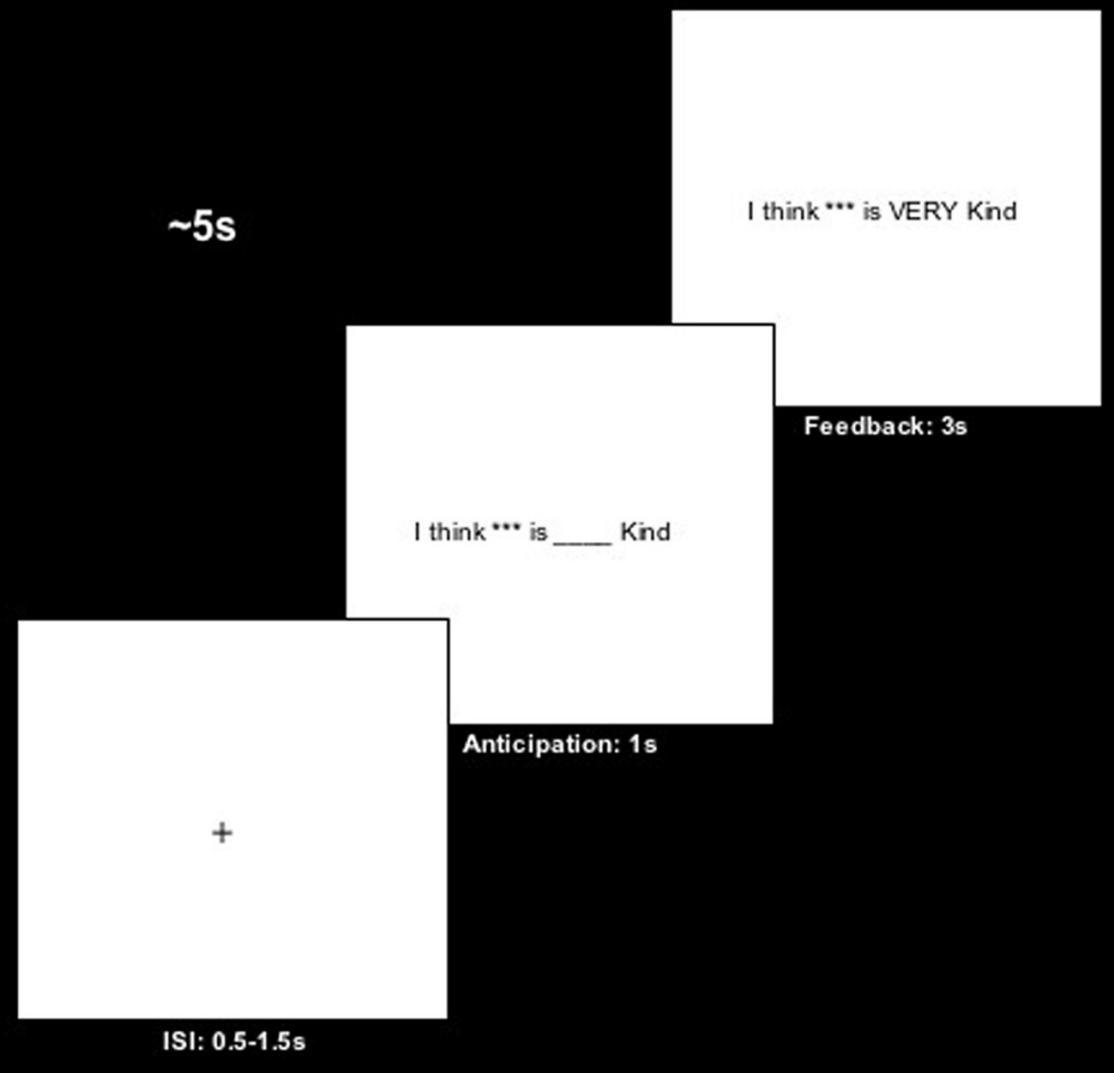
**Schematic depiction of trials within blocks.** Trials were composed of a.5–1.5 s interstimulus interval (ISI; *M* = 1 s). This was followed by a 1s anticipatory event, which presented participants with statements reflecting the trait their partner's appraised them on, excluding their partner's actual appraisal. Finally, participants were presented with an adjective associated with partner's appraisals of them (“feedback”) in a 3 s event.

The remaining 90 trials were presented as part of the actual task. Each displayed one item taken from the appraisal questionnaires. Items were placed in sentences phrased as though participants' partners were directly reporting them. For example, for the “kind” item, participants would see, “I think (Participants' Name) is VERY kind.” Furthermore, trials were comprised of three parts: an uniformly sampled interstimulus interval (ISI) or “jitter” lasting 0.5–1.5 s, an anticipatory event [e.g., I think (Participants' Name) is _____ kind”] lasting 1.0 s, and “feedback” [e.g., I think (Participants' Name) is VERY kind”] lasting 3.0 s (see Figure 1). Additionally, of the 90 trials, 48 were confirmatory, 21 were positive violations, and 21 were negative violations. Trials were nested into blocks of 10, which were counterbalanced across the task. Blocks, like trials, varied with respect to confirmation or violation and were counterbalanced across the task. Confirmatory blocks were composed of eight confirmation trials, one positive expectation violation trial, and one negative violation trial. Positive and negative expectation violation blocks were composed of six violation trials and four confirmation trials. This design was adopted for two primary reasons: (1) to ensure that across the task and violation blocks, participants' expectations across the task remained centered around their responses to the reflected appraisal questionnaire thereby preserving the efficacy of stimulus presented as confirmatory feedback or a violation of a priori expectations; (2) to examine the differential impact of expectation violations in either positive or negative directions on social reward processing and task-related reports of attachment anxiety and trust (see below).

Interspersed between the blocks were randomized questions that prompted participants to reflect on and report affect related to experiences of romantic passion. Each question separately asked for participants' reports of feeling enthusiastic/excitement (i.e., “How much ENTHUSIASM or EXCITEMENT do you feel regarding your partner's responses *right now*?”) and feeling upset/anxious (i.e., “How much UPSET or ANXIETY do you feel regarding your partner's responses *right now*?”). Participants were given five seconds to rate their feelings for each question on a four-point Likert scale [responses: 0 (“None”), 1 (“A Little”), 2 (“A Lot”), 3 (“A Great Deal”)], using a button box. After participants completed the task, they were asked to report on their contemporaneous feelings of relationship-specific attachment anxiety (Brennan et al., 1998) and partner-specific trust (Rempel et al., 1985) in a post-task questionnaire, using measures identical to those in the intake questionnaires.

## Results

### Behavioral responses

Of the 17 participants in the MRI study, 16 completed both pre- and post-task measures of relationship-specific attachment anxiety and partner-specific trust. One additional outlier evidenced high levels of anxiety in pre- and post-task anxiety measures (more than 4 SD from the mean) and was excluded from analysis, leaving 15 participants included in these analyses. Paired-samples *t*-tests revealed no significant differences between pre- (*M* = 5.65, *SD* = 0.67) and post-task measures of trust (*M* = 5.43, *SD* = 0.94) [*t*_(14)_ = 1.37, *p* = 0.19]. However, participants' post-task attachment anxiety reports (*M* = 2.91, *SD* = 1.34) were significantly greater than their pre-task reports (*M* = 2.19, *SD* = 1.19) [*t*_(14)_ = 3.34, *p* < 0.05]. This increase from pre- to post-task anxiety remained significant when the aforementioned outlier was included (see “Appendix”). Moreover, the differences between pre- and post-task attachment anxiety and trust were inversely associated (*r* = −0.90, *p* < 0.001)—increases in attachment anxiety (relationship uncertainty) across the task accompanied decreases in trust (relationship certainty).

Across the task, reports of affect related to romantic passion varied by block type. *Post-hoc* comparisons based on multiple One-Way, repeated measures ANOVAs suggested that participants reported feeling more enthusiasm/excitement following positive violation blocks than either negative violation blocks (Δ*M* = 0.99, *SE* = 0.13, *p* < 0.001) or confirmatory blocks (Δ*M* = 0.51, *SE* = 0.12, *p* < 0.01). Likewise, participants reported feeling more upset/anxious following negative violation blocks than either positive violation blocks (Δ*M* = 0.83, *SE* = 0.11, *p* < 0.001) or confirmatory blocks (Δ*M* = 0.49, *SE* = 0.16, *p* < 0.05) (see Table 1).

**Table 1:** **Marginal means for affect measures by block type**.

**Block Type**	**Positive**	**Confirmatory**	**Negative**
**MEASURE**			
Enthusiasm/Excitement	3.40^a,1^	2.89^b,2^	2.41^a,b,3^
Upset/Anxiety	1.09^a,1^	1.43^a,b,2^	1.93^a,b,3^

Identical superscript letters indicate statistically significant differences across rows. Identical superscript numbers indicate statistically significant differences across columns.

Participants reported more attachment anxiety following the prediction-error task, which involved 42 violations of expected feedback (out of 100). And, although participants did not report less trust for their partner following the task, decreases in pre- to post-task trust were associated with increases in attachment anxiety. This indicates that the task challenged participant's expectations about their partner's sentiments toward them, engendering a sense of uncertainty about their relationship, and evoking reactions that coincide with such uncertainty.

### Neuroimaging data

**Figure 2:**
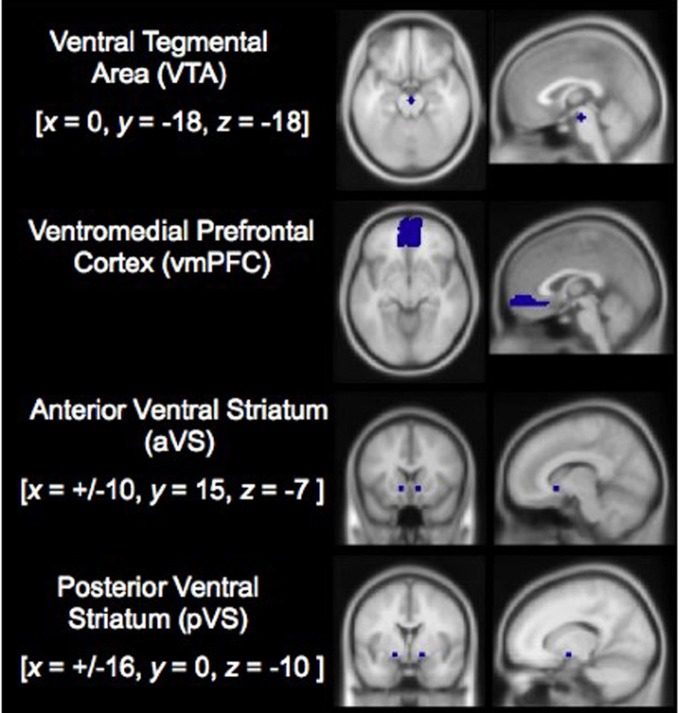
**Independent, a priori defined regions of interest used in analyses.** Note: *x*, *y*, and *z* refer to MNI coordinates indicating the centers of mass for each ROI in left-right, anterior-posterior, and superior-inferior dimensions.

**Figure 3:**
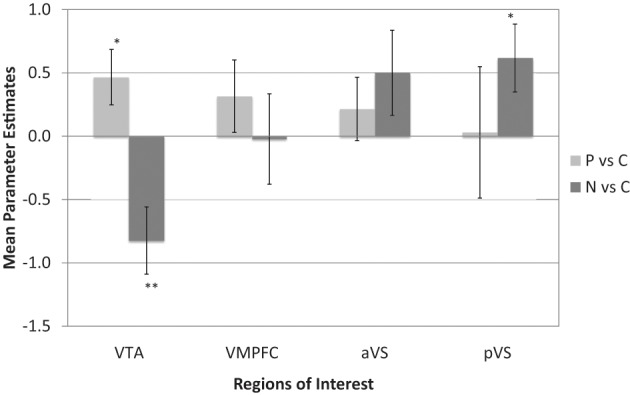
**Mean differences in ROI parameters, by contrast.** ^*^ = *p* < 0.05, ^**^ = *p* < 0.01. Note: **P vs C** = Positive Violations – Confirmations; **N vs C** = Negative Violations – Confirmations.

All analyses reported here relied on *a priori* region of interest (ROI) contrasts between events using the MarsBaR toolbox for SPM (Version 0.41; Brett et al., 2002). ROIs were specified in advance for reward system areas: the aVS and pVS, the VTA, and the vmPFC (see Figure 2; see “Appendix” for ROI specification). Statistical analyses were first conducted by way of contrasts comparing confirmatory trials with violation trials (both positive and negative combined), and then by comparing confirmatory trial with both positive and negative violation trials separately. In contrasts between confirmatory and combined violation trials, no significant differences were observed across the four ROIs, save for a marginal effect suggesting increased aVS activity in during violation trials compared to confirmatory trials [*t*_(16)_ = 1.48, *p* = 0.08]. However, finer comparisons between confirmatory trials and each violation trial types (positive and negative) revealed that responses in the VTA were enhanced during positive violation trials relative to confirmatory trials [*t*_(16)_ = 2.14, *p* < 0.025] and diminished during negative violation trials relative to confirmatory trials [*t*_(16)_ = −3.10, *p* < 0.01]. There were no significant differences in vmPFC activity during either positive violation trials compared to confirmatory trials [*t*_(16)_ = 1.11, *p* = 0.14], or negative violation trials compared to confirmatory trials [*t*_(16)_ = −0.06, *p* = 0.52]. Activity in the aVS was not significant during positive violation trials relative to confirmatory trials [*t*_(16)_ = 0.86, *p* = 0.20], but exhibited marginally significant increases during negative violation trials compared to confirmatory trials [*t*_(16)_ = 1.49, *p* = 0.08]. Finally, the pVS demonstrated significant increases during negative violation trials compared to confirmatory trials [*t*_(16)_ = 2.30, *p* < 0.025], but not in positive violation trials relative to confirmatory trials [*t*_(16)_ = 0.06, *p* = 0.48] (see Figure 3).

Overall, the observed pattern suggests that VTA activation discriminated between positive violation, negative violation, and confirmatory social feedback, in a specific pattern consistent with prior research on prediction-error in the reward system. Likewise, the pattern of pVS activity corresponds with that of Seymour and colleagues (2007) research, which found increased activation at similar coordinates for negative prediction-errors in the context of economic losses relative to gains (*x* = −16, *y* = 0, *z* = −10) (see also Delgado et al., 2008). This suggests that negative violations of expectations elicited prediction-error signals in independently defined, *a priori* ROIs within the VTA and pVS.

### Effects of positivity/negativity

We next investigated an alternative hypothesis—whether ROI activity across trials of each type (i.e., positive violation, confirmation, negative violation) was an artifact of the magnitude with which the task stimuli were valenced (positivity or negativity) rather than being modulated by the confirmatory or non-confirmatory (expecation violation) nature of the stimuli. First, we assigned numerical values to task stimuli associated with each trial, indicating their valence (e.g., a little = 1, very = 4, exceedingly = 7). These values (i.e., valence) were then modeled as a linear parametric modulator of hemodynamic response for each trial type. We examined whether ROI activity was linearly associated with the valence of task stimuli. If ROI activity was an artifact of the positivity or negativity of the stimuli, then it should be linearly associated with ROI activity across all trial types, regardless of their confirmatory or non-confirmatory nature. However, we found no significant or marginal linear association between ROI activity and the magnitude with which stimuli were valenced, in either positive violation or confirmatory trials. Valence was inversely associated with both vmPFC activity [*t*_(17)_ = −3.50, *p* < 0.01] and pVS activity [*t*_(17)_ = −1.70, *p* = 0.05], but only for negative violation trials. This suggests that VTA ROI activity resembling prediction-error signals was not an artifact of the positivity or negativity of the task stimuli alone, but driven by the confirmatory or non-confirmatory nature of the stimuli and the directionality (positive or negative) of expectation violations. It should be noted that confirmatory stimuli were not “neutral,” but included the same range of positivity or negativity as was presented in either violation trial-type. Additionally, pVS activity was *selectively* modulated within negative violation trials; activity was both sensitive to non-confirmatory, aversive stimuli, and selectively tracked the degree of deviation from expectations, or loss. This is consistent with prior findings that such activity tracks aversive prediction-errors in the context of economic loss (Seymour et al., 2007, 2012; see Delgado et al., 2008 for review).

### Associations between self-report and fMRI responses

**Figure 4:**
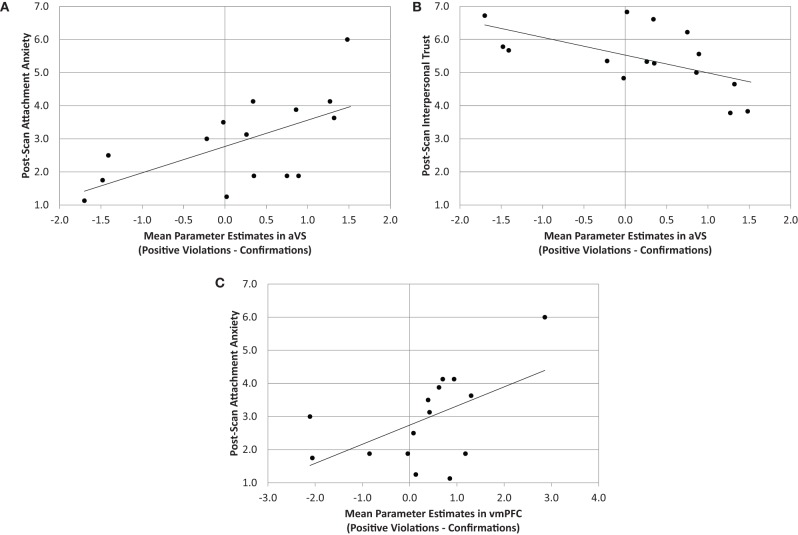
**Associations between ROI parameters and post-task measures.** Significant associations between: **(A)** aVS activity and post-task attachment anxiety; **(B)** aVS activity and post-task interpersonal trust; **(C)** vmPFC activity and post-task attachment anxiety. All activity is from positive violation trials relative to confirmatory trials.

Parameter estimates from the analyses above (i.e., differences between confirmation and both types of violation trials) were correlated with task-related affect and measures of both partner-specific attachment security and partner-specific trust. These analyses included 15 of the 17 total participants—one was excluded due to failure to complete both pre- and post-test attachment measures and another due to statistically anomolous reports of high anxiety in both pre- and post-task reports of attachment (see above; see also “Appendix”). No significant associations emerged between task-related affect and ROI activity. Also, there were no significant associations between pre-post task difference scores on measures of attachment and trust. However, ROI activity was related to post-task measures of both attachment and trust. Post-task attachment anxiety was positively associated with activity in the vmPFC (*r* = 0.54, *p* < 0.05) and aVS (*r* = 0.61, *p* < 0.05) in positive violation trials compared to confirmatory trials (see Figure 4). Furthermore, activity in the aVS from the same contrast was negatively associated with post-task reports of trust (*r* = −0.58, *p* < 0.05). These associations remained statistically reliable even with the inclusion of outliers; aVS associations with anxiety and trust remained significant, and vmPFC associations with anxiety were marginal. Put differently, greater activity in these regions during the receipt of unexpectedly positive feedback was associated with greater partner-specific attachment anxiety after the task, and less partner-specific trust.

## Discussion

Previous research demonstrates that the reward system is sensitive to social rewards, but such research generally utilizes economic games played between strangers, social evaluatory paradigms involving simulated interactions with peers, or studies of romantically involved participants that lack any interactive component. With a sample of real romantic partners and utilizing an adapted gain-loss paradigm (after Seymour et al., 2007), we investigated reward system processing of stimuli that either confirmed or violated expectations of social-reward, and whether reward system processing under these conditions was in turn related to task-related attachment anxiety and trust directed toward relationship partners. Our results suggest that violation of *a priori* social-reward expectations within the context of pre-existing social relationships elicits a prediction-error-like signal in dey reward-system regions of interest (i.e., VTA, striatum). Similar to those discovered in other paradigms investigating social reward. Unexpected gains and losses in partners' positive esteem for the self and relationship modulated reward-system activity consistent with other research on prediction-error; compared to confirmatory trials, positive expectation violation trials yielded activation in the VTA, while negative expectation violation trials yielded deactivation in the VTA and activation in the pVS. Though activity in the aVS did not exhibit prediction-error-like modulation in response to non-confirmatory stimuli (see Pessiglione et al., 2006; cf. Robinson et al., 2010), the patterns of activation/deactivation we find in the VTA are remarkably consistent with prior findings in reward system prediction-error in both social (Behrens et al., 2008; Jones et al., 2011; Lin et al., 2012) and non-social paradigms (Abler et al., 2006; Schultz, 2006; D'Ardenne et al., 2009). In this respect, our results both extend findings from previous studies and lend additional external validity to processes implicated by previous research—reward system activity may play an important role in day-to-day social cognition within interactions between actual relationship partners.

While the contribution of the pVS to prediction-error processing in the reward system is less clear than that of the VTA or anterior aspects of the ventral striatum, its activity might reflect serotonergic processes that modulate dopaminergic activity in the anterio-medial ventral striatum under conditions of reward-related loss (Seymour et al., 2007, 2012; cf. Delgado et al., 2008). Our findings lend support of the role in this area to reward processing; like Seymour et al. (2007, 2012) we used gain/loss paradigm for studying reward system prediction-error, rather than a dichotomous outcome paradigms (reward/reward omission, reward/punishment, social inclusion/exclusion). We replicate their findings of Seymour et al. (2007) and extend them to the context of social reward, finding that task-related valence inversely modulated pVS activity, but only in the context of negative expectation violation. Given (1) that in previous research differentiating anterior from posterior VS processes in the context economic reward and loss, pVS activity was selective for non-positive, loss-related aversive prediction-error, (2) our own findings in the pVS and the VTA, and (3) that our findings in the VTA activity were not an artifact of the positivity or negativity associated with task stimuli, but rather whether or not stimuli were positive or negative *deviations* from expected outcomes, our results indicate a prediction-error-like signal in the context of pure social feedback within existing relationships.

Previous research on social reward has not yet made direct linkage between social attachment-related mental representations in real social relationships, and the affect that accompanies changes in these representations. While we found significant increases in attachment anxiety and decreases in trust across the task, we did not find that pre-post task difference scores on measures of attachment and trust were related to BOLD signals—perhaps due to a subtle manipulation and small effect sizes in these comparisons. However, we find that post-task reports of relationship-specific attachment anxiety and partner-specific trust did covary with reward system activation in theoretically meaningful ways. First, attachment anxiety was associated with increased reward system activation in positive violation trials relative to confirmatory trials, in regions that are strongly associated with appetitive goal-pursuit (aVS, vmPFC; see Depue and Morrone-Strupinsky, 2005). This is a meaningful association given that attachment anxiety is an appetitive representation that encompasses an uncertainty about relational outcomes, a compulsive drive for closeness with partners, and intense positive and negative experiences of love (Eastwick and Finkel, 2008). Although it seems paradoxical that unexpected reward-related gains could promote anxiety, this is precisely what attachment theory would predict; attachment anxiety represents an uncertainty about relational outcomes and the extent to which partners reciprocate romantic sentiment (Shaver and Mikulincer, 2006), but does not exclusively manifest as negative experiences. Rather, it is related to compulsive partner proximity seeking (appetitive behavior) and therefore may stem from both positive and negative experiences arising from either unexpected gains or losses in perceptions of interpersonal closeness (romantic passion: Baumeister and Bratslavsky, 1999; Eastwick and Finkel, 2008). Second, we find that post-task reports of trust were *inversely* associated with aVS activation in positive violation trials relative to confirmatory trials. Conceptually, trust runs opposed to attachment anxiety. It is based upon notions of predictability, dependability, and faith—certainty that partners will fulfill our needs (Rempel et al., 1985). In this respect, these findings compliment our attachment findings, suggesting that aVS activation is positively associated with feelings of uncertainty in relationships (attachment anxiety), but inversely associated with feelings of certainty in relationships (trust). Moreover, given that aVS activation in positive violation trials relative to confirmatory trials was related to outcomes (i.e., increased anxiety, decreased trust), task-related variations in self-report data and neural modulation were more likely driven by errors in prediction rather than by the positivity or negativity of the stimuli, alone. If the later were the case, we would expect a pattern in opposition to the one we found.

Collectively, our findings supplement previous research suggesting that the reward system might not just monitor social reward outcomes but, through its integration with the medial prefrontal cortex, motor cortex, and limbic system, may be involved in learning and developing explicit, partner-specific representations of attachment security and trust, as well as behavioral strategies in service of achieving social needs for understanding, self-validation, and care (see Reis and Patrick, 1996; Reis et al., 2004; see also Ortigue and Bianchi-Demicheli, 2008). Additionally, our findings illustrate a central process thought to underlie social affiliation: self-verification, a tendency for people to seek social ties to confirm their self-perceptions, fulfilling a desire to maintain a sense of predictability and control (see Swann et al., 1990, 1992). In this respect, even self-enhancing feedback may be threatening if it is unexpected or inconsistent with prevailing beliefs about the self (e.g., positive violations). Our findings are consistent with this perspective, as we found that unexpected positive feedback is tied to both momentary activation of the reward system and anxiety-laden cognitions (attachment anxiety).

Our findings reveal a number of fertile avenues for future investigation. First, previous studies find that prediction-error events with respect to economic outcomes promote better recognition of contemporaneously presented stimuli (see Adcock et al., 2006). Future studies could attempt to replicate these findings in the context of social reward and examine associations between recognition, task-related affect, and task-related changes in attachment representations. Additionally, future studies could examine the extent to which different proportions of confirmatory stimuli, relative to stimuli that violate expectations, alter reward-system activity and subsequent representations. Such studies might provide an opportunity to examine how subcortical activity in social reward paradigms contribute to dynamic changes in anticipatory activity in cortico-representational areas (e.g., vmPFC) and whether such change is linked with change in reports of attachment security and trust.

### Conflict of interest statement

The authors declare that the research was conducted in the absence of any commercial or financial relationships that could be construed as a potential conflict of interest.

## Appendix

### Methods

#### General-laboratory session

Confirmatory statements presented to participants, on average, were associated with the modifier “Very” (mean scale value: 5.3, range = 2.65). Positive violations were generally associated with the modifier “Extremely” (mean scale value: 6.4, range = 1.24) and negative violations associated with “Fairly” (mean scale value: 3.4, range = 2.24).

#### Deception

When participants were told that they would receive “feedback” from their partners' questionnaires, they were told that this was contingent on additional consent from them and their partner to release this information. Both partners were asked for consent to release their questionnaire data to their partners, simultaneously. They were asked for consent while separated and told that their decision was their own, independent of that of their partners. Therefore, they were not told whether their partner consented prior to their decision. All participants included in this report provided such consent (along with their partner). These efforts were made to help ensure the believability of the deception. Throughout the course of this study only one participant did not provide consent. To preserve their confidentiality in this decision, their partner was told that the server shut down and questionnaire data could not be accessed and that the experiment could not proceed as a result (Ethernet cables were disconnected to provide authentic illustrations of this to the partner). Both the participant and their partner were paid for their time and debriefed with respect to the procedures they completed.

#### Debriefing and deception interview

Following scanning and post-scan questionnaires, participants were individually asked whether they had any questions about their experiences. At this time no participants volunteered questions, expressed immediate concerns, or questioned the authenticity of task stimuli. Participants were then reunited with their partners, and were debriefed again, with their partners. During this time, the full purpose of the experiment was revealed and the extent of the deception was described. Participants were told that the only “feedback” that was authentic (actually from their partners' questionnaires) was that which was presented in the trials incorporating audio recordings from their partners (training trials). Furthermore, participants were told that this feedback was specially chosen because it confirmed their own expectations and thus provided them no new information past what they believed was already true. Experimenters made very clear, at this point, that *none* of the other “feedback” was authentic, but was contrived for the experiment. Following this, participants were informally interviewed as to their feelings during the task and whether they were suspicious about the authenticity of the task stimuli during the task. Some fMRI participants reported that they had felt upset about some of the feedback during the task, but when asked, did not indicate that they still felt upset following the debriefing. Every fMRI participant reported that there were a number of times that they felt surprised by some of the task stimuli (consistent with the aims of the task), but no participant reported that they harbored suspicion about the authenticity of the stimuli across the task. After all participants' questions were answered and the nature of the experiment was fully revealed, participants were probed as to whether they felt hurt or upset at their partners. No participants reported as such, but were nonetheless provided information regarding psychological counseling services and given contact info for the lead experimenter (JCP). Finally, since the completion of the experiment, no adverse events have been reported by participants.

#### Neuroimaging

Functional neuroimaging data were acquired on a Siemens Trio 3T scanner housed at the UCLA Ahmanson-Lovelace Brain Mapping Center. Each participant was scanned using a high-resolution structural T2-weighted echo-planar image (spin-echo, TR = 4000 ms, TE 54 ms, matrix size 128 × 128, FOV = 20 cm, 36 axial slices, 1.56-mm in-plane resolution, and 3-mm thick), which was acquired coplanar with functional scans. Participants completed the prediction-error task across four functional runs. The first was a training run (4:50 s) consisting of only confirmatory events (these data were not included in analyses). The remaining three functional runs (8:48 s) each incorporated one of each block type and three rest periods (fixation cross-hairs) lasting 14 s each (gradient-echo, TR = 2000 ms, TE = 25 ms, flip angle = 90°, matrix size 64 × 64, FOV = 20 cm, 36 axial slices, 3.125-mm in-plane resolution, and 3-mm thick). Images were prescribed along the anterior commissure/posterior commissure line.

#### fMRI data analysis

Imaging data were analyzed using statistical parametric modeling (SPM5; Wellcome Department of Cognitive Neurology, Institute of Neurology, London, UK). Images were realigned, temporally corrected, normalized, and smoothed with an 8 mm Gaussian kernel, full width at half maximum. Analyses relied on the general linear model in an event-related analysis. Effects at each voxel were estimated using linear contrasts to compare specific regional activity. For each contrast, participants' imaging data were aggregated for single subject analysis and for group level analysis according to the random effects model in SPM5.

Negative and positive expectation violation trials were modeled as events, as were confirmatory trials from confirmatory blocks. Confirmatory events from positive and negative expectation violation blocks were not modeled as confirmatory events. Epochs for these events consisted of the feedback periods for each trial (see Figure 1). Inter-stimulus intervals were modeled with rest periods and the 1-s anticipatory periods beginning each trial were separately modeled from event epochs.

Custom ROIs were built using the WFU Pickatlas toolbox for SPM5 (Maldjian et al., 2003), based upon considerations from previously reported localizations for the VTA (8 mm diameter sphere at *x* = 0, *y* = −18, and *z* = −18; after de Greck, et al., 2008) and posterior VS (bilateral 8 mm diameter spheres at *x* = ± 16, *y* = 0, and *z* = −10; after Seymour et al., 2007) on similar tasks. Also, two ROIs were anatomically specified, one for the anterior VS/nucleus accumbens, specified at the ventromedial aspect of the caudate head and putamen junction (bilateral 8 mm diameter spheres at *x* = ± 10, *y* = 15, and *z* = −7; based on the Automated Anatomical Atlas (AAL), Tzourio-Mazoyer, et al., 2002), and one for the bilateral vmPFC, constructed from predefined, preloaded AAL shapes in the WFU Pickatlas (Maldjian et al., 2003). All coordinates are reported in MNI format.

### Results

#### Self-report responses

Analyses comparing pre- and post- task measures of relationship-specific attachment anxiety and partner-specific trust included 15 of the 16 participants with complete pre- and post- task data. One outlier evidenced high levels of anxiety in pre- and post-task anxiety measures (more than 4 SD from the mean) and was not included in this analysis. However, pre- and post-task comparisons of anxiety remained significant [*t*_(15)_ = 2.28, *p* < 0.05] while comparisons for trust were insignificant [*t*_(15)_ = 1.21, *p* = 0.25] with the inclusion of the participant.

#### Associations between self-report and fMRI responses

Correlational analyses examining associations between neural activity and post-task reports of anxiety and trust included 15 of the 17 participants. The outlying anxiety and trust data points that were previously excluded (see above) were not included in this analysis, though aVS associations with anxiety (*r* = 0.65, *p* < 0.01) and trust (*r* = 0.61, *p* < 0.05) remained significant when these data points were included and vmPFC associations with anxiety were marginally significant (*r* = 0.45, *p* = 0.08).
